# Proteome Analysis of the Soybean Nodule Phosphorus Response Mechanism and Characterization of Stress-Induced Ribosome Structural and Protein Expression Changes

**DOI:** 10.3389/fpls.2022.908889

**Published:** 2022-06-09

**Authors:** Yubo Yao, Hongmei Yuan, Guangwen Wu, Chunmei Ma, Zhenping Gong

**Affiliations:** ^1^Heilongjiang Academy of Agricultural Sciences Postdoctoral Programme, Harbin, China; ^2^Institute of Industrial Crops, Heilongjiang Academy of Agricultural Sciences, Harbin, China; ^3^College of Agriculture, Northeast Agricultural University, Harbin, China

**Keywords:** proteome, soybean, phosphorus, root nodule, nitrogen fixation

## Abstract

In agroecosystems, a plant-usable form of nitrogen is mainly generated by legume-based biological nitrogen fixation, a process that requires phosphorus (P) as an essential nutrient. To investigate the physiological mechanism whereby phosphorus influences soybean nodule nitrogen fixation, soybean root nodules were exposed to four phosphate levels: 1 mg/L (P stress), 11 mg/L (P stress), 31 mg/L (Normal P), and 61 mg/L (High P) then proteome analysis of nodules was conducted to identify phosphorus-associated proteome changes. We found that phosphorus stress-induced ribosomal protein structural changes were associated with altered key root nodule protein synthesis profiles. Importantly, up-regulated expression of peroxidase was observed as an important phosphorus stress-induced nitrogen fixation-associated adaptation that supported two nodule-associated activities: scavenging of reactive oxygen species (ROS) and cell wall growth. In addition, phosphorus transporter (PT) and purple acid phosphatase (PAPs) were up-regulated that regulated phosphorus transport and utilization to maintain phosphorus balance and nitrogen fixation function in phosphorus-stressed root nodules.

## Introduction

Biological nitrogen fixation plays an extremely important role in the natural nitrogen cycle by providing an indispensable and sustainable source of reduced nitrogen that supports survival and reproduction of all organisms within the biosphere. Soybean [Glycine max (L) Merr], an important grain, source of oilseed and ingredient of animal feed, is one of the most widely cultivated legume crops. In fact, the soybean crop accounts for about 68% of legume crop production and 57% of oilseed production globally (Herridge et al., 2008). Agriculturally, the rhizobia-legume symbiosis nitrogen fixation system, the most efficient natural nitrogen fixation system, fixes about 40 million tons of pure nitrogen per year, which is equivalent to about 65% of overall agricultural biological nitrogen fixation capacity (Lai, 2019). In Brazil, biological nitrogen fixation is responsible for generating 70–85% of usable nitrogen required by soybeans, which is equivalent to 70–250 kg of nitrogen per hectare, an amount that basically highlights the self-sufficiency of soybeans to generate most of the nitrogen they need (Alves et al., 2003; Peoples et al., 2009). Therefore, maximizing the efficiency of biological nitrogen fixation during soybean cultivation can provide a green and pollution-free nitrogen source to support high-yield crop growth that reduces use of chemical fertilizers and protects the ecological environment.

Phosphorus is the second most important nutrient element needed for plant growth, due to its role as a key component of many cellular macromolecules (e.g., nucleic acids and phospholipids) and its energy storage role as a component of ATP. More specifically, phosphorus participates in cellular activities such as energy-related photosynthesis, respiration, DNA transcription and translation, nutrient absorption and other biological reactions. These activities are indispensable for plant growth, development and metabolic activities that, in turn, are essential for obtaining high crop yield and quality (Cordell and White, 2014; Yang et al., 2020). Notably, N_2_ fixation is a process that supports phosphorus-requiring energy conversion/consumption and lipid and protein synthesis activities that play extremely important roles in legume root nodule formation and nitrogen fixation (Mullen et al., 1988; Gentili and Huss-Danell, 2003; Zhang et al., 2014; Taliman et al., 2019). The important role of phosphorus has been revealed through results of numerous studies that have shown significant inhibition of root nodule formation and growth under low-phosphorus stress conditions (Miao et al., 2009; Vardien et al., 2014). In addition, these phenotypic changes are accompanied by significantly reduced nodular nitrogenase activity and nitrogen fixation capacity that markedly hinder plant growth (Chaudhary et al., 2008; Hernández et al., 2009; Valentine et al., 2017). Consequently, these changes have been shown to be associated with low Pi availability that, in turn, triggers dramatic changes in gene and protein expression in plants (Lin et al., 2010; Zhang et al., 2014).

In order to adapt to low-phosphorus stress, plants have evolved various mechanisms to tolerate low-phosphorus conditions that involve the induction of huge changes in gene and protein expression patterns (Lin et al., 2010; Zhang et al., 2014; O’Rourke et al., 2020; Zhao et al., 2021). Importantly, the phosphate starvation response (PHR) transcription factor is the central regulatory factor that directly or indirectly regulates activities of low-phosphorus response genes (phosphate stress induced genes, PSI genes) within the low-phosphorus regulatory network (Nilsson et al., 2007; Zhou et al., 2008; Lu et al., 2020). Under low-phosphorus conditions, significant increases were observed in expression of phosphoenolpyruvate phosphatase in bean nodules (Barsgaz et al., 2012) and purple acid phosphatases (PAPs) and high-affinity phosphate transporter GmPT5 (Qin et al., 2012) in soybean nodules. Therefore, investigations of effects of phosphorus stress on soybean nodule protein metabolism should help to uncover physiological mechanisms used by soybean to maintain nitrogen fixation capacity under phosphorus stress conditions. In the present study, we report proteome differences in root nodules exposed to different concentrations of phosphorus using a tandem mass tag (TMT)-based quantitative proteomics-based approach.

## Materials and Methods

### Plant Growth and Treatment

Soybean cultivar SN14 (120 days or 2450 C in heat units from seeding to maturity) was used as experiment material. Soybean plants were cultured in sand medium in pots. The culture conditions and P levels used here [1, 11, 31, 61 mg/L and denoted by P1 (P-stress), P11 (P-stress), P31 (normal-P), P61 (high-P)] are the same as those described by Yao et al. (2018).

Before the vegetative cotyledon stage (VC, unfolded cotyledons), the plants were only supplied with 500 ml of distilled water once a day. From VC to V_3_ (third trifoliate leafs) stages, P31 nutrient solution was supplied. Starting from V_3_ stage and thereafter, different P treatments were supplied in 500 ml nutrient solution once a day for 15 days.

Rhizobium was inoculated when the opposite true leaves fully opened, the method was as follows: soybean nodules collected in the previous year and stored in the refrigerator were ground and the ground material was added to the nutrient solution. Soybean plants were inoculated for five consecutive days to ensure that every plant was inoculated.

### Sample Preparation

Detailed steps and methods of protein extraction, BCA assay, acetone precipitation, redissolve, reduction, alkylation, protein digestion, TMT labeling, SDC cleanup, peptide desalting and high-pH pre-fractionation were described in Supplementary Material 1. The obtained samples were used for proteomic analysis.

### Nano Liquid Chromatography-Mass Spectrometry/Mass Spectrometry (LC-MS/MS) Analysis

For each sample, 1 μg of total peptides were separated and analyzed with a nano-ultra high performance liquid chromatog (UPLC) (EASY- nLC1200) coupled to a Q Exactive HFX Orbitrap instrument (Thermo Fisher Scientific) with a nano-electrospray ion source. Separation was performed using a reversed-phase column (100 μm ID × 15 cm, Reprosil Pur 120 C18 AQ, 1.9 μm, Dr. Maisch). Mobile phases were H_2_O with 0.1% FA, 2% ACN (phase A) and 80% ACN, 0.1% FA (phase B). Separation of sample was executed with a 90 min gradient at 300 nL/min flow rate. Gradient B: 2–5% for 2 min, 5–22% for 68 min, 22–45% for 16 min, 45–95% for 2 min, 95% for 2 min.

Data dependent acquisition (DDA) was performed in profile and positive mode with Orbitrap analyzer at a resolution of 120,000 (@200 m/z) and m/z range of 350–1600 for MS1; For MS2, the resolution was set to 45 k with a fixed first mass of 110 m/z. The automatic gain control (AGC) target for MS1 was set to 3E6 with max IT 30 ms, and 1E5 for MS2 with max IT 96 ms. The top 20 most intense ions were fragmented by HCD with normalized collision energy (NCE) of 32%, and isolation window of 0.7 m/z. The dynamic exclusion time window was 45 s, single charged peaks and peaks with charge exceeding 6 were excluded from the DDA procedure.

### Proteome Discoverer Database Search

Vendor’s raw MS files were processed using Proteome Discoverer (PD) software (Version 2.4.0.305) and the built-in Sequest HT search engine. MS spectra lists were searched against their species-level UniProt FASTA databases (uniprot-Glycine max-3847-2021-3.fasta), with Carbamidomethyl (C), TMT 6 plex (K) and TMT 6 plex (N-term)as a fixed modification and Oxi- dation (M) and Acetyl (Protein N-term) as variable modifications. Trypsin was used as proteases. A maximum of 2 missed cleavage(s) was allowed. The false discovery rate (FDR) was set to 0.01 for both PSM and peptide levels. Peptide identification was performed with an initial precursor mass deviation of up to 10 ppm and a fragment mass deviation of 0.02 Da. Unique peptide and Razor peptide were used for protein quantification and total peptide amount for normalization. All the other parameters were reserved as default.

## Results

### Differentially Expressed Proteins in Soybean Nodules Exposed to Different Levels of Phosphorus

**FIGURE 1:**
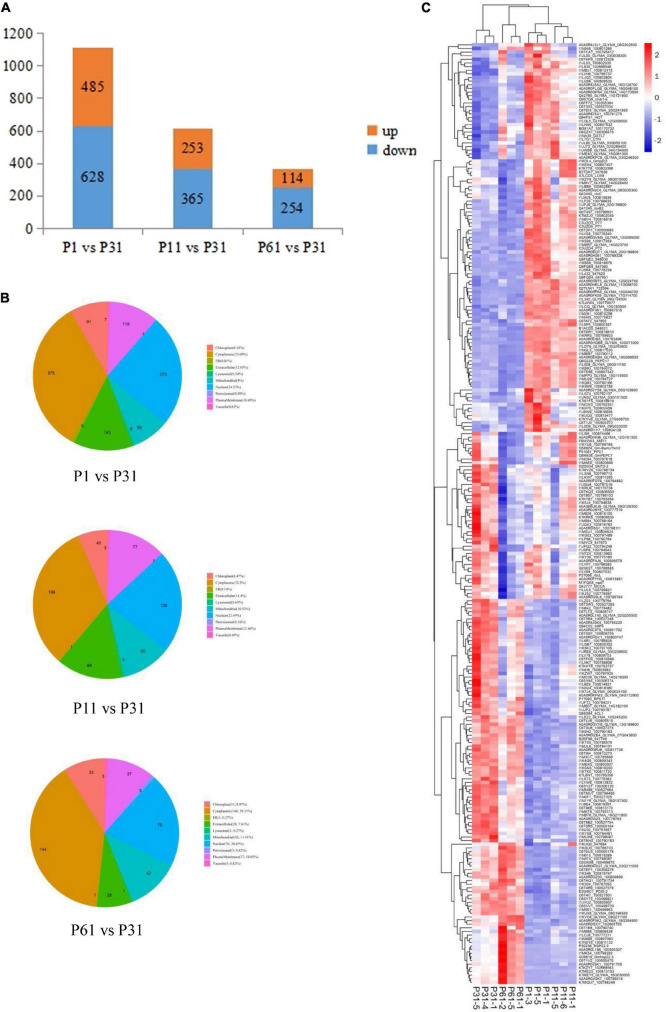
Analysis of the DEPs. **(A)** Number of DEPs, up-regulated and down- regulated expression between P1 vs. P31, P11 vs. P31, P61 vs. P31; **(B)** Pie chart for subcellular localization analysis of DEPs; **(C)** Heatmap clustering analysis of DEPs. The color blocks at different positions represent the relative expression levels of the proteins at the corresponding positions, red represents the high expression level, and blue represents the low expression level.

After soybean plants were exposed to different levels of phosphate, a total of 8,016 proteins were detected, of which Differentially Expressed Proteins (DEPs) were identified based on criteria that included unique peptide ≥ 1, fold change ≥ 1.5 or ≤ 0.67 and *P*-value < 0.05 indicating significantly up-regulated and down-regulated proteins. Ultimately, comparisons of paired P treatment group data (P1 vs. P31, P11 vs. P31 and P61 vs. P31) led to identification of 1113, 618, and 368 DEPs, respectively, of which 485/628, 253/365, and 114/254 of DEPs were up-regulated/down-regulated (Figure 1A), respectively. Subcellular localization analysis showed that more than 50% of DEPs were detected within the cytoplasm or nucleus. Moreover, a greater proportion of DEPs were located extracellularly in root nodules of plants exposed to phosphorus stress conditions than in nodules of unstressed plants (Figure 1B). Results of heatmap clustering analysis of DEPs [red indicates higher protein expression and blue indicates lower protein expression as compared to that of the P31 sample (Figure 1C)] along with associated gene names, accession numbers and descriptions of DEPs are provided in Supplementary Table 1. Notably, these results indicated that a greater number of DEPs were functionally associated with metabolic processes as compared to other process types, thus indicating that metabolic processes enabled phosphorus-stressed plants to adapt to and mitigate phosphorus stress-induced effects on root nodule nitrogen fixation.

### Gene Ontology Functional Annotation

**FIGURE 2:**
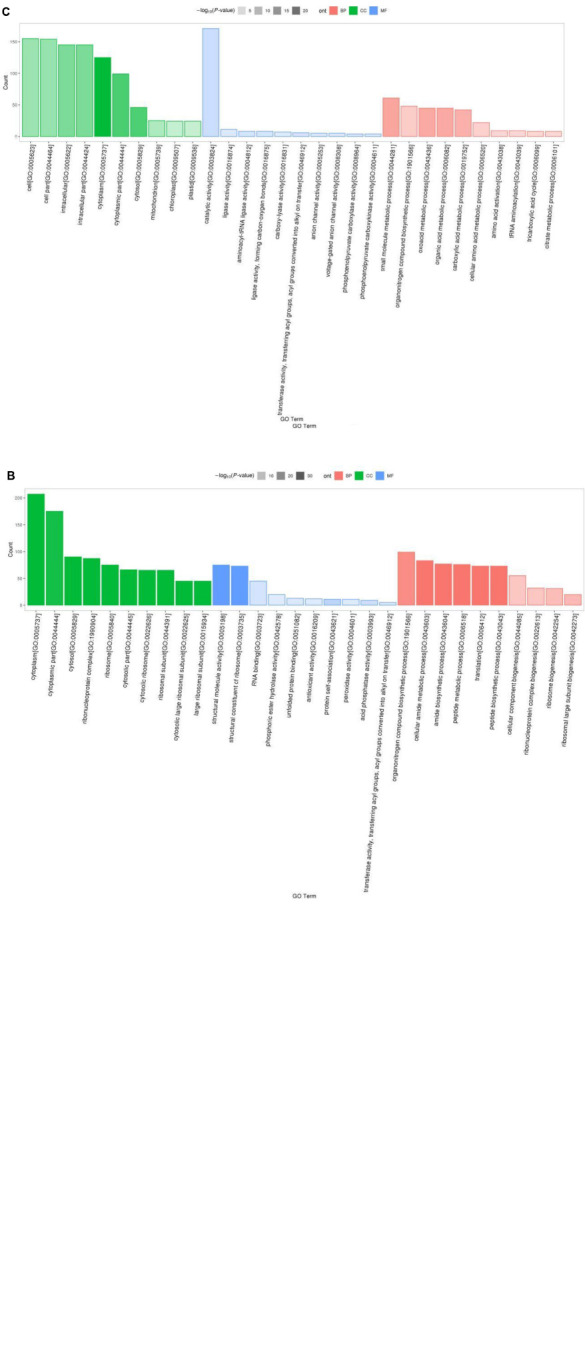
GO enrichment analysis classification histogram of DEPs. The ordinate is the number of DEPs mapped, red indicates BP annotation information, green indicates CC annotation information, blue indicates MF annotation information, transparency indicates *p-*value size, the darker the color, the smaller the *p*-value. **(A)** P1 vs. P31; **(B)** P11 vs. P31; **(C)** P61 vs. P31.

Results of Gene Ontology (GO) analysis revealed that 1113 detected DEPs for P1 vs. P31 were enriched for 466 GO terms, of which 61 terms were related to cellular component, 120 to molecular function and 285 to biological process GO categories. The top 10 GO functional terms for P1 vs. P31 DEPs shown in Figure 2A. With regard to cellular component GO terms, most were related to cytosolic large ribosomal subunit, cytosolic ribosome, cytosolic part, large ribosomal subunit and ribosomal subunit terms. With regard to molecular function GO terms, most were related to structural constituent of ribosome, structural molecule activity, unfolded protein binding, RNA binding and protein self-association terms. With regard to biological process GO terms, most were related to translation, peptide biosynthetic process, amide biosynthetic process, peptide metabolic process and cellular amide metabolic process terms.

The top 10 significantly enriched GO functional terms for P11 vs. P31 DEPs are shown in Figure 2B. Overall, the 618 DEPs were associated with 353 GO terms, of which 43 terms belonged to cellular component, 97 to molecular function and 213 to biological process GO categories. With regard to cellular component GO terms, most were related to cytosolic large ribosomal subunit, cytosolic ribosome, large ribosomal subunit, cytosolic part and ribosomal subunit terms. With regard to molecular function GO terms, most were related to structural constituent of ribosome, structural molecule activity, protein self-association, AP activity and RNA binding terms. With regard to biological process GO terms, most were related to translation, peptide biosynthetic process, amide biosynthetic process, peptide metabolic process and cellular amide metabolic process terms.

The top 10 significantly enriched GO functional terms for P61 vs. P31 DEPs are shown in Figure 2C. Overall, the 368 DEPs were enriched for 366 GO terms, of which 57 terms belonged to cellular component, 79 to molecular function and 230 to biological process GO categories. With regard to cellular component terms, most were related to cytoplasm, cytoplasmic part, cytosol, intracellular, and intracellular part terms. With regard to molecular function terms, most were related to catalytic activity, transferase activity, aminoacyl-tRNA ligase activity, ligase activity, forming carbon-oxygen bonds and phosphoenolpyruvate carboxylase activity terms. With regard to biological process terms, most were related to small molecule metabolic process, oxoacid metabolic process, organic acid metabolic process, carboxylic acid metabolic process and organonitrogen compound biosynthetic process terms.

### Kyoto Encyclopedia of Genes and Genomes Pathway Enrichment Analysis

Results of Kyoto Encyclopedia of Genes and Genomes (KEGG) pathway enrichment analysis highlighted main biochemical metabolic pathways and signal transduction pathways associated with DEPs, with enriched pathways and numbers of DEPs associated with each pathway differing among the three P level-based pairwise comparisons. DEPs of P1 vs. P31 were significantly enriched for 6 metabolic pathways that were related to ribosome, protein processing in endoplasmic reticulum, linoleic acid metabolism, synthesis and degradation of ketone bodies, amino sugar and nucleotide sugar metabolism and ubiquinone and other terpenoid-quinone biosynthesis. DEPs of P11 vs. P31 were significantly enriched for 5 metabolic pathways that were related to ribosome, protein processing in endoplasmic reticulum, riboflavin metabolism, amino sugar and nucleotide sugar metabolism and galactose metabolism. DEPs of P61 vs. P31 were mainly enriched for 12 metabolic pathways that were related to carbon metabolism, nitrogen metabolism, sugar metabolism and secondary metabolism (Table 1). Taken together, the results of KEGG pathway enrichment analysis of DEPs for all phosphorus-stress-based comparison groups exhibited similarities, although results obtained for P61 were significantly different from results that were obtained for P1 and P11 groups.

**TABLE 1:** Differences of metabolic pathways.

Treatments	KEGG ID	Description	Number of differential proteins
P1 vs. P31	gmx03010	Ribosome	97
	gmx04141	Protein processing in endoplasmic reticulum	42
	gmx00591	Linoleic acid metabolism	8
	gmx00072	Synthesis and degradation of ketone bodies	3
	gmx00520	Amino sugar and nucleotide sugar metabolism	17
	gmx00130	Ubiquinone and other terpenoid-quinone biosynthesis	8
P11 vs. P31	gmx03010	Ribosome	69
	gmx04141	Protein processing in endoplasmic reticulum	20
	gmx00740	Riboflavin metabolism	4
	gmx00520	Amino sugar and nucleotide sugar metabolism	12
	gmx00052	Galactose metabolism	6
P61 vs. P31	gmx00520	Amino sugar and nucleotide sugar metabolism	14
	gmx01210	2-Oxocarboxylic acid metabolism	7
	gmx01230	Biosynthesis of amino acids	15
	gmx00020	Citrate cycle (TCA cycle)	6
	gmx00970	Aminoacyl-tRNA biosynthesis	7
	gmx01110	Biosynthesis of secondary metabolites	40
	gmx01200	Carbon metabolism	13
	gmx01100	Metabolic pathways	63
	gmx00261	Monobactam biosynthesis	2
	gmx00630	Glyoxylate and dicarboxylate metabolism	5
	gmx00920	Sulfur metabolism	3
	gmx00100	Steroid biosynthesis	3

## Discussion

Phosphorus is an essential nutrient element for plant photosynthesis, as well as for synthesis and transport of nucleic acids, nucleoproteins and carbohydrates (Chen and Xue, 2012). Therefore, phosphorus directly affects growth and development of nodules, while also supporting nitrogenase activity and nitrogen fixation function of legume crops (Israel, 1993; Schulze et al., 2006; Wang et al., 2009; Chen et al., 2011; Qin et al., 2012). In fact, results of several studies suggest that phosphorus deficiency leads to significantly reduced nodule development and nitrogen fixation activity even in soybean plants with sufficient numbers of nodules (Miao et al., 2007). Our previous research showed that low-phosphorus stress significantly inhibited nitrogen accumulation and nodule nitrogen fixation (Yao et al., 2018). Based on those previous results, here proteomic analysis of root nodules was carried out to obtain in-depth knowledge related to the soybean root nodule nitrogen fixation response in order to identify changes associated with phosphorus nutritional status from a molecular perspective.

### Root Nodule Ribosome Responses to Phosphorus Stress

**FIGURE 3:**
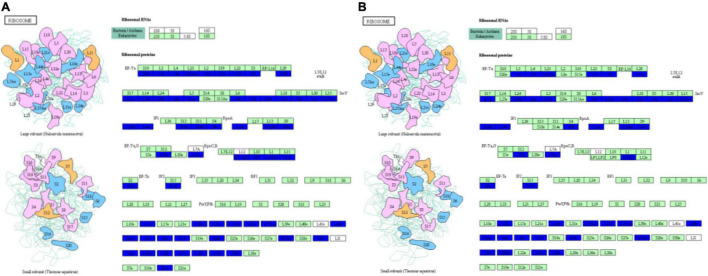
Changes in protein conformation and large and small ribosomal subunits structural under p-stress. **(A)** P1 vs. P31; **(B)** P11 vs. P31.

Ribosomes, which are molecular factories that carry out protein synthesis, are sophisticated molecular machines that decode the genetic blueprint present within mRNA to allow proper assembly of amino acids carried by tRNAs during synthesis of protein molecules (Bashan and Yonath, 2008). In eukaryotic cells, three distinct types of ribosomes are found in different cellular compartments: in the cytoplasm, in mitochondria and in plant chloroplasts. Although all three types of ribosomes possess large and small subunits, they differ from one another with regard to ribosomal RNA (rRNA) and protein sequences (Robles and Quesada, 2017). Cell growth, which corresponds to an increase in cell mass, requires prodigious numbers of ribosomes that are generated *via* a highly regulated cellular process that determines the growth capacity of a cell (Lempiainen and Shore, 2009). Moreover, results of several studies have shown that molecular interactions occurring between ribosomes and aminoacyl-tRNAs trigger significant ribosomal and tRNA conformational changes during mRNA decoding (Pape et al., 1999) that lead to ribosomal domain closure (Ogle and Ramakrishnan, 2005; Demeshkina et al., 2012) and tRNA structural distortion (Schuette et al., 2009; Schmeing and Ramakrishnan, 2009; Villa et al., 2009; Demeshkina et al., 2012). In this study, we found that phosphorus stress led to structural and protein conformation changes in both large and small ribosomal subunits (Figure 3), with decreasing phosphorus level associated with increasing down-regulation of expression of ribosomal proteins (Supplementary Table 2). At the same time, results of metabolomic analysis indicated that aminoacyl-tRNA biosynthesis in soybean nodules also changed under phosphorus stress (Supplementary Tables 3, 4). Taken together, these results suggest that phosphorus stress triggered structural changes in proteins within the ribosome that subsequently affected synthesis of root nodule proteins such as peroxidase, pi transporter and other proteins related to soybean nodule formation and nitrogen fixation. However, mechanisms underlying ribosome structural changes and protein interactions with aminoacyl-tRNAs are unknown, warranting further study.

### Root Nodule Peroxidase-Associated Responses to Phosphorus Stress

Intracellular reactive oxygen species (ROS) are maintained in a dynamic balance between production and elimination during plant growth and development. When plants are subjected to biotic or abiotic stresses, disruption of ROS homeostasis occurs that leads to excessive ROS accumulation that causes irreversible damage to nucleic acids, proteins and lipids that eventually culminates in cell death (Gechev et al., 2005; Garg and Manchanda, 2009). Long-term evolutionary processes have led to the incorporation of antioxidant defense systems in plants that rely on enzymes that include peroxidases (PODs), glutathione peroxidase (GPx), etc. These defense systems remove peroxides to alleviate stress-induced damage, while also maintaining a relatively stable internal environment (Zhao et al., 2006; Ma et al., 2012). Peroxidase not only plays a role in stress resistance, but also frequently participates in various physiological processes, such as cell wall growth, cell wall modification, lignin formation, auxin catabolism, resistance to pathogen infection, seed germination and senescence (Gazaryan et al., 1999; Passardi et al., 2004; Passardi et al., 2005; Almagro et al., 2009; Cosio and Dunand, 2009; Shigeto and Tsutsumi, 2016). Here we observed up-regulated expression of peroxidases in root nodules under phosphorus stress (P1 and P11), with the number of distinct peroxidases that were expressed increasing with increasing phosphorus stress level (Supplementary Table 5). And ROS (SOD and POD) activity in leaves increased under phosphorus stress (Supplementary Table 6). Meanwhile, peroxidases also play a key role in the polymerization of lignin monomers (Zhao et al., 2013), a process that leads to cessation of plant cell growth (Vanholme et al., 2019) that may ultimately influence nodule volume. Here we found that the effect of phosphorus stress on nodule number was smaller than its effect on nodule volume, as based on determinations of nodule numbers and weights after 15 days of phosphorus stress (Table 2). Therefore, up-regulation of peroxidase expression in root nodules appeared to be an important mechanism for maintaining soybean nodule nitrogen fixation capability during soybean adaptation to phosphorus stress. On the one hand, these results suggest that peroxidase scavenges harmful substances that are produced in response to stress, such as ROS. On the other hand, increased peroxidase levels influenced cell wall growth by promoting lignin synthesis that stopped cell growth, resulting in decreased nodule volume.

**TABLE 2:** The nodules number, volume and changes compared to P31.

Treatments	Nodules number		Nodules weight	
	(per plant)	(%)	(mg/nodule)	(%)
P1	118.67 ± 9.82b	–25.53 ± 2.83c	2.49 ± 0.13b	–54.24 ± 2.03b
P11	130.67 ± 4.05b	–17.98 ± 1.21b	2.53 ± 0.07b	–53.47 ± 0.94b
P31	159.33 ± 7.31a	–	5.44 ± 0.24a	–
P61	167.67 ± 3.84a	5.24 ± 0.14a	5.95 ± 0.20a	9.37 ± 0.44a

*Vertical comparison. The data are represented as mean values ± standard error (with four replicates). Values with the same letters are not significantly different at the 5% level.*

### Phosphate Stress-Induced Phosphate Transporter and Acid Phosphatase Activity Changes

Root nodules rely on functions of two main pathways for acquiring phosphorus: a direct absorption pathway, as well as an indirect Pi transport pathway that involves Pi translocation from the root of the host plant to the root nodule (Al-Niemi and Mcdermott, 1998; Li et al., 2021). Phosphorus transporter (PT) and acid phosphatase (AP), especially PAPs participate in regulation of phosphorus balance in soybean nodules. Importantly, PT pathways (especially high-affinity PT pathways) play key roles in phosphorus uptake and transport (Xue, 2018). For example, results of several soybean studies have shown that the high-affinity PT pathway that is active within vascular bundles of roots and nodules controls root-to-nodule phosphorus transport (Qin et al., 2012; Chen, 2017; Li, 2018). In addition, this pathway also regulates soybean nodule formation and growth, especially under P-limiting conditions.

By contrast, enzymatic activities of PAPs, which are broad-spectrum hydrolases, have been shown to be increased in roots of plants in low-phosphorus environments, where these enzymes promote activation and absorption of organic phosphorus within the rhizosphere. Moreover, increased enzyme activity *in vivo* can promote reuse of organophosphorus in old leaves (Bozzo et al., 2002; Li et al., 2002; Wang et al., 2009; Hur et al., 2010), while enhanced synthesis and secretion of PAPs by plant roots is viewed as a main mechanism used by plants to adapt to low-phosphorus stress conditions (Zhou M Y. et al., 2021), as reported for GmPAP14 in Arabidopsis (Zhou Y. et al., 2021). Specifically, expression of GmPAP14 was induced under low-phosphorus conditions, while overexpression of this enzyme significantly improved organic phosphorus utilization efficiency (Zhou Y. et al., 2021). Overexpression of GmPAP12 resulted in higher nodule number, fresh weight, and nitrogenase activity under low phosphorus stress, indicating that GmPAP12 may promote P utilization in soybean nodules under low P stress, which thus played an important role in nodulation and biological nitrogen fixation (Wang et al., 2020).

In this study, three high-affinity PT (up-regulated) were found to be associated with soybean plant responses to phosphorus stress. Notably, these pathways were found to be associated with plasma membranes and were not detected in plants of the P61 group. Meanwhile, in P1 vs. P31, 1 AP and 8 PAPs were up-regulated, while in P11 vs. P31, 1 AP and 6 PAPs were up-regulated (Supplementary Table 7). Measurements of phosphorus contents in shoots and root nodules under phosphorus stress revealed that phosphorus content changes in root nodules were significantly less marked than corresponding changes in shoots, thus indicating that phosphorus content of root nodules remained relatively stable (Supplementary Table 8). Taken together, these results showed that phosphorus stress-induced up-regulation of expression of PT pathway enzymes and PAPs in root nodules led to enhanced regulation of phosphorus transport and reuse activities that ultimately maintained root nodule phosphorus balance and preserved nitrogen fixation activity.

## Data Availability Statement

The data presented in the study are deposited in the ProteomeXchange repository, accession number PXD033875.

## Author Contributions

YY were responsible for distribution of materials integral to the findings presented in this article, conceived the original screening and research plans, project and wrote the article with contributions of all the authors, performed most of the experiments and analyzed the data, and agreed to serve as the author responsible for contact and ensures communication. ZG, YY, and CM supervised the experiments. HY and GW provided technical assistance. All authors contributed to the article and approved the submitted version.

## Conflict of Interest

The authors declare that the research was conducted in the absence of any commercial or financial relationships that could be construed as a potential conflict of interest.

## Publisher’s Note

All claims expressed in this article are solely those of the authors and do not necessarily represent those of their affiliated organizations, or those of the publisher, the editors and the reviewers. Any product that may be evaluated in this article, or claim that may be made by its manufacturer, is not guaranteed or endorsed by the publisher.

## Supplementary Material

Supplementary Table 1 Details of DEPs.

Supplementary Table 2 Changes of ribosomal protein under phosphorus stress.

Supplementary Table 3 Amino acid and derivatives differences in root nodules under phosphorus stress.

Supplementary Table 4 Up-regulated and down-regulated of amino acid in aminoacyl-tRNA biosynthesis pathway.

Supplementary Table 5 Peroxidase differences under phosphorus stress.

Supplementary Table 6 Changes of ROS in leaves under different phosphorus levels.

Supplementary Table 7 Acid phosphatase and purple acid phosphatase differences under phosphorus stress.

Supplementary Table 8 Changes of phosphorus content in shoots and nodules under different phosphorus stress.
