# Supplementary material for: Proteome Analysis of the Soybean Nodule Phosphorus Response Mechanism and Characterization of Stress-Induced Ribosome Structural and Protein Expression Changes
Source: Front Plant Sci. 2022 Jun 9;13:908889. doi: 10.3389/fpls.2022.908889 (PMC9218819; doi:10.3389/fpls.2022.908889)
Supplement: Supplementary Table 6 — Changes of ROS in leaves under different phosphorus levels. [file Table_6.docx]

Sample Preparation

1 Protein extraction

Take 1/2 of the sample into a new EP tube, add an appropriate amount of phenol extract and protease inhibitor, and grind at low temperature for 5 minutes. Add an equal volume of Tris (PH 8.0) Equilibrate the saturated phenol solution, react at 4°C for 30 minutes, shaking once every 5 minutes. Centrifuge at 7,200 g for 15 min at 4 °C to collect the phenol upper layer. Add pre-chilled 0.1 M ammonium acetate-methanol solution and precipitate overnight at -20 °C. Centrifuge at 4 °C of 12000 g for 10 min, collect the precipitate, add pre-cooled methanol to wash and mix, centrifuge at 4 °C of 12000 g. Collect the precipitate for 10 minutes and repeat it once. Repeat the previous step twice with acetone instead of methanol to fully remove methanol. Centrifuge at 12000 g for 10 min at 4 °C, collect the precipitate, and air dry for 1 min. Dissolve the dried powder in the sample lysis solution by pipetting and mixing, and ultrasonicate for 5 minutes. Centrifuge at 12000 g for 10 min at 4 °C, and transfer the supernatant to a new ep tube.

2 BCA assay

Pipette BCA working solution to 96-well plate, 200 uL per well, 7 standard points, 1 blank. Add 20 uL samples (diluted by the corresponding multiple) or standard protein (BSA). Shake at 37°C for 30 min, and measure the absorbance at 562 nm. Fit the standard curve according to the standard protein, and calculate the protein concentration of the corresponding sample.

3 Acetone precipitation

Take 100 ug total protein for each sample and dilute to 1 mg/mL with H_2_O. Acetone is pre-cooled to -20 °C, and 5 times the volume of acetone is added to the sample. After mixing, it will precipitate overnight at 20 °C. Centrifuge at 12,000 rpm, 4 °C for 10 minutes, and carefully remove the supernatant. Add 200 uL pre-cooled 80 % acetone to rinse the pellet twice, centrifuge at 12,000 rpm, and carefully remove the supernatant.

4 Redissolve & reduction & alkylation

Add 100 uL protein recombination solution and sonicate the protein in a water bath for 5 min to dissolve the protein precipitate.

Add DTT to 5mM and incubate at 55 °C with shaking for 10 min to reduce disulfide bonds. Cool the sample to room temperature, add IAA to 10 mM, and react for 15 minutes in the dark to alkylate the reduced disulfide bonds.

5 Protein digestion

Dissolve Trypsin to 0.5 ug/uL in Resuspension buffer, and incubate at room temperature for 5 min. Mix the Trypsin with the sample thoroughly at the ratio of Trypsin: protein = 1:50. After simple centrifugation, incubate overnight at 37 °C with shaking at 1000 rpm.

6 TMT labeling

Centrifuge the sample at high speed for 10 minutes, and transfer the same amount of protein to a new ep tube. Follow the instructions of Thermo Company's TMT labeling kit for labeling. Mix equal amounts of each group of labeled samples.

7 SDC cleanup

Add TFA to the mixed sample (final concentration 2 %, pH <2), mix well to precipitate SDC. Centrifuge at high speed for 10 minutes and transfer the supernatant to a new EP tube. Add n*100 uL 2 % TFA, mix well, centrifuge at 13,000 rpm for 10 min, and extract the co-precipitated peptides (weight Re-extract 2 times). Combine the supernatant fractions several times, centrifuge at high speed for 10 minutes, and transfer the supernatant to a new EP tube to obtain a labeled peptide sample.

8 Peptide desalting

Add 1mL Buffer C to the C18 cartridge to activate, and make all the solution flow slowly into the centrifuge tube. Add 1mL Buffer A to equilibrate, and make all the solution flow slowly into the centrifuge tube. Add the sample supernatant, let all the solution flow slowly into the centrifuge tube, and collect the effluent (FT). Add 1mL Buffer A and rinse twice to make all the solution flow slowly into the centrifuge tube. Add 400 uL Buffer B for elution, use a pipette to transfer the elution hydraulic pressure to the new EP tube (E1). The effluent FT desalination is repeated once (E2), and the two eluates (E1+E2) are combined. Dry under vacuum at 4 °C overnight.

9 High-pH pre-fractionation

After lyophilization, the peptide samples were reconstituted to 50 uL with mobile phase A, and separated under alkaline conditions using RPUPLC. Column: 150 mm×2.1 mm (waters, XBridge BEH C18 XP Column); mobile phase A: 10 mM ammonium acetate aqueous solution, pH = 10; mobile phase B: 10 mM ammonium acetate, 10 % H_2_O, 90 % ACN, pH =10. Liquid gradient 60 min, mobile phase B: 5 % for 2 min, 5-30 % for 40 min, 30-40 % for 10 min, 40-90 % for 4 min, 90 % for 2 min, 2 % for 2 min . One component is collected every 1 min and collected in a cycle. There are 12 components in total. After vacuum drying, they are frozen and stored at 80 °C.
